# Facile, High Quality Sequencing of Bacterial Genomes from Small Amounts of DNA

**DOI:** 10.1155/2014/434575

**Published:** 2014-11-13

**Authors:** Momchilo Vuyisich, Ayesha Arefin, Karen Davenport, Shihai Feng, Cheryl Gleasner, Kim McMurry, Beverly Parson-Quintana, Jennifer Price, Matthew Scholz, Patrick Chain

**Affiliations:** ^1^Los Alamos National Laboratory, P.O. Box 1663, MS M888, Los Alamos, NM 87545, USA; ^2^Parkview Medical Center School for Medical Laboratory Science, 400 West 16th Street, Pueblo, CO 81003, USA

## Abstract

Sequencing bacterial genomes has traditionally required large amounts of genomic DNA (~1 *μ*g). There have been few studies to determine the effects of the input DNA amount or library preparation method on the quality of sequencing data. Several new commercially available library preparation methods enable shotgun sequencing from as little as 1 ng of input DNA. In this study, we evaluated the NEBNext Ultra library preparation reagents for sequencing bacterial genomes. We have evaluated the utility of NEBNext Ultra for resequencing and *de novo* assembly of four bacterial genomes and compared its performance with the TruSeq library preparation kit. The NEBNext Ultra reagents enable high quality resequencing and *de novo* assembly of a variety of bacterial genomes when using 100 ng of input genomic DNA. For the two most challenging genomes (*Burkholderia* spp.), which have the highest GC content and are the longest, we also show that the quality of both resequencing and *de novo* assembly is not decreased when only 10 ng of input genomic DNA is used.

## 1. Introduction

The rapid improvement in quality, quantity, and cost of next generation sequencing (NGS) has resulted in commensurate improvements in analysis techniques. For bacteria, high throughput sequencing has become a routine task. The availability of kits for library preparation, rapid and high content sequencing, and mature data analysis pipelines for genome resequencing and assembly had drastically reduced costs and improved reliability of these results. The commoditization of bacterial genome sequencing has led to more complex applications: clinical and agricultural diagnostics [1–4], outbreak detection and monitoring [5–7], human health studies [8, 9], biocatalysis [10, 11], environmental studies [12], and many others [13, 14].

For NGS platforms, current sequencing technologies require that sequencing adapters be ligated to DNA fragments before sequencing is possible. Ligation of adapters to (typically small) DNA fragments is an inefficient process, generating ligated hybrids from only a small fraction of targeted DNA molecules. This limitation in turn increases the required DNA input, with the only goal being to generate sufficient numbers of ligated fragments to allow sequencing. Typical library preparation methods require large amounts (~1 *μ*g) at high concentrations (>25 ng/mL) of DNA for successful library generation, limiting the types of samples that can be sequenced reliably.

Existing library preparation methods have several reported limitations. These include high variability of evenness and completeness of genome coverage as a function of %GC content, input DNA quantities, and sequencing technology [15–19]. These impact the amount of sequencing data required and the quality of genome assembly and analysis.

Several library preparation kits that require 1–100 ng of input DNA are now available (New England Biolabs' NEBNext, Illumina's TruSeq Nano, Bioo Scientific's NEXTflex, NuGEN's Ovation Ultralow, etc.). This paper details the results of evaluation of the utility of the NEBNext Ultra library preparation kits for both resequencing and assembly of several bacterial genomes. We compare the evenness and completeness of coverage between NEBNext Ultra and Illumina TruSeq kits for bacterial genomes of varying size and %GC content. Our findings indicate that low DNA input amounts are sufficient to generate high quality sequencing data that can be used for genome resequencing or* de novo* assembly (if combined with long fragment data).

## 2. Materials and Methods

### 2.1. Overview

We sequenced three different bacterial species with various genome lengths (from 5.4 Mb to 6.7 Mb) and containing various %GC contents (from 35% to 68%). Standard input DNA amounts were 100 ng, approximately 10x lower than the required amount for the Illumina TruSeq kit and 10x higher than the minimum DNA inputs per NEBNext Ultra manual specifications. The most challenging (longest genome and highest GC content) bacterial genomes (*Burkholderia* A and B) were also sequenced with minimal DNA inputs (10 ng). All samples were sequenced on the Illumina HiSeq platform using 2 × 100 bp chemistry. Data analyses consisted of read-mapping the short fragment data to reference genomes using BWA (Burrows-Wheeler Alignment). These data were also combined with long insert mate pair data to evaluate their utility for* de novo* assembly of the bacterial genomes.

### 2.2. Bacterial Strains and Genomic DNA Preparation

Genomic DNA from* Bacillus anthracis* (strain Sterne 34F2) was isolated from a log phase culture using the MO-BIO UltraClean microbial DNA isolation kit. The* Escherichia coli* strain 2009EL-2050 and genomic DNA purification have been previously described [20].* Burkholderia thailandensis* A (strain E254, accession numbers CP004381 and CP004382) and* Burkholderia thailandensis* B (strain USAMRU Malaysia #20, accession numbers CP004383 and CP004384) are previously reported strains, and DNA was provided by Dr. Paul Keim's group (sequences to be published in Spring 2014). The integrity of all genomic DNA samples was evaluated using agarose gels and their quantity measured with PicoGreen reagents on a Qubit 2.0 instrument.

### 2.3. Library Preparation (Figure S1)

NEBNext Ultra library preparation protocol consists of several enzymatic and two purification steps, one of which is used for size selection of library fragments. Genomic DNA samples were sheared in 55 *μ*L of TLE buffer (10 mM Tris, 0.1 mM EDTA, pH 8) using Covaris E220 with the following settings: duty cycle 10%, intensity 5, cycle 200, and time 100 sec. After shearing, two enzymatic steps (end preparation and adapter ligation) are performed in the same tube, followed by size selection of the library fragments using a double AMPure cleanup. First AMPure step used 0.4x sample volume of beads and the supernatant was transferred to a clean tube. The second AMPure step used 0.2x sample volume of beads. Selected library fragments were amplified with barcoded primers (10–12 PCR cycles) and purified one more time with AMPure beads (0.5x bead volume) (see Supplementary Material available online at http://dx.doi.org/10.1155/2014/434575).

### 2.4. Library Quality Control, Quantification, and Sequencing

NEBNext libraries were analyzed using Bioanalyzer 2100 and DNA 1000 or DNA high sensitivity chips, to quantify the library size and assess the level of adapter-dimer and primer-dimer contamination. Libraries were quantified using Illumina library qPCR quantification kits from KAPA Biosystems and sequenced on either the Illumina MiSeq or Illumina HiSeq.

The Illumina data from this study were trimmed to remove any ambiguous bases; any reads shorter than 70 bp after trimming and the corresponding read pairs were discarded. The total number of reads per sample ranged from 6.2 million to 47.8 million before trimming. All data had read lengths of 151 bp with one exception which had read lengths of 101 bp. After trimming, the average read lengths were reduced by less than 3.5% for all samples. The data for each sample were normalized to 70x coverage of the genome after trimming. The average number of reads with a quality greater than Q20 after trimming and normalization ranged from 61% of the total reads to 97% of the total reads. The total number of reads, the number of reads with quality greater than Q20, and the average read lengths before and after trimming for each sample can be found in Table S1. The assemblies were compared to the reference genomes to consider insertion/deletion errors and rearrangements using an in-house Perl script.

### 2.5. Mapping of Reads to Reference Genomes

For read-mapping, all trimmed reads from each preparation were used. Burrows-Wheeler Alignment (BWA) mapping tool was used, combined with SAMtools and in-house Perl scripts for coverage and insert size analysis [21, 22]. For base coverage we used BWA global alignment option with default parameters. BWA global alignment only reports the best alignment based on score calculated by a set of parameters. If a read has several possible best alignment spots, BWA randomly assigns the read to one spot. All reads mapped to contigs were used to calculate base coverage. For insert size calculation, only properly paired reads (read pair on the same contig and with correct orientation) were used. We report the mean, standard deviation, the minimum, and maximum of the insert size distribution for all short fragment libraries. We utilized three thresholds for reporting coverage: 0%, 1%, and 10% of mean fold coverage.

### 2.6. Genome Coverage

Calculation of evenness of coverage was performed by calculating the average and standard deviation of coverage across nonoverlapping 10 kbp fragments of the finished genome. Evenness for each fragment was calculated as 1 − (standard deviation of coverage/coverage). All data points (genomic and plasmid coverage, where appropriate) were used to generate box and whisker plots in IBM's statistics program SPSS.

### 2.7. Assembly Methods

Two deBruijn graph assembly tools were used to evaluate the quality of the short fragment data for the purpose of assembling high quality genomes. IDBA uses only paired reads from short fragment Illumina libraries [23]. Paired reads were randomly selected (*in silico*) from each sample to generate libraries of approximately 70-fold genome coverage for each sample, in order to normalize the data. The only exception was the* E. coli* sample prepared with the TruSeq kit, for which only 61-fold coverage was available. Each data set was assembled with IDBA, version 1.1.0.

The 70-fold short fragment Illumina data were combined with previously sequenced long insert mate pair data generated by 454. The 454 data had an average insert size of 8 kbps and provided 7- to 8-fold base coverage, with the exception again for the* E. coli* samples, which had approximately 3.5-fold coverage. The combined data were assembled with Allpaths, version 44837 [24]. The 454 data were used without trimming or data reduction in the Allpaths assembly.

## 3. Results and Discussion

### 3.1. Library Preparation (Figure S1 and Table S1)

The library preparation protocol, as described in Section 2, yields average insert sizes of ~270 ± 15 bps (average library sizes of ~400 ± 15 bps) that are optimal for either 2 × 100 or 2 × 150 bp sequencing on Illumina platforms. Different insert sizes can easily be obtained by adjusting the size selection step (ratio of DNA solution to AMPure beads) as recommended by the manufacturer. It is not necessary to adjust the shearing step, as the sheared DNA produced by Covaris has a very broad size distribution. NEBNext library process provides very consistent results in terms of library size and concentration, even when performed for the very first time.

Prior to normalization and sequencing, samples were analyzed using Qubit (PicoGreen-based method), Bioanalyzer 2100, and quantitative real-time PCR (qPCR, KAPA Biosystems). When the libraries are quantified by qPCR, accurate normalization and clustering was achieved. Unfortunately, this was not the case when molar library concentrations were obtained with Qubit and Bioanalyzer data only (without qPCR). Therefore, we recommend that qPCR library quantification is routinely performed. Sequencing was performed on either Illumina HiSeq (2 × 100 bp) or Illumina MiSeq (2 × 150 bp).

### 3.2. Evenness of Coverage

**Figure 1:**
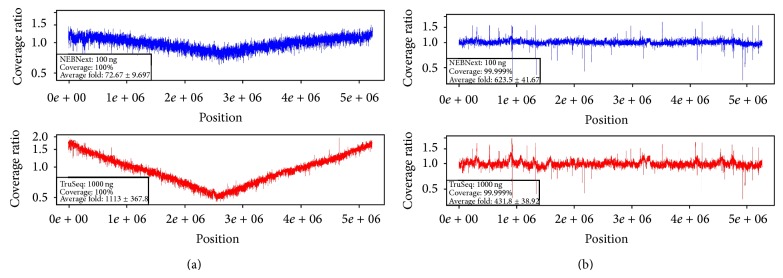
Evenness of coverage for* B. anthracis* (a) and* E. coli* (b) genome sequencing using NEBNext library preparation with 100 ng DNA input (top graph) and TruSeq library preparation with 1,000 ng DNA input (bottom graph). Plots are normalized by the average coverage in each figure.

**Figure 2:**
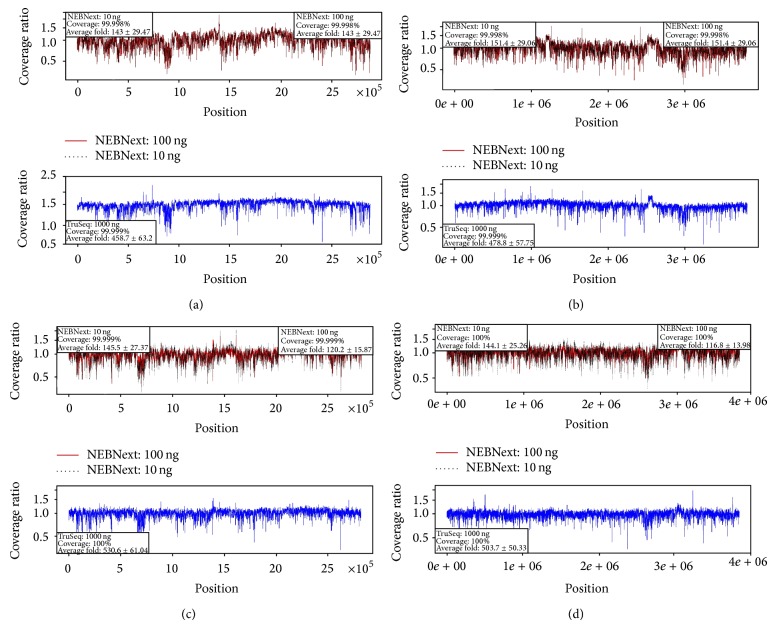
Evenness of coverage plots for* B. thailandensis* A ((a) is chromosome 1 and (b) is chromosome 2) and* B. thailandensis* B ((c) is chromosome 1 and (d) is chromosome 2) genome sequencing. Top graph within each panel shows NEBNext library preparation data with 10 ng or 100 ng DNA input (black color is for 10 ng samples and red color is for 100 ng samples). Bottom graph within each panel shows TruSeq library preparation data with 1,000 ng DNA input. Plots are normalized by the average coverage in each figure.

Figure 1 (*B. anthracis* and* E. coli*) and Figure 2 (*B. thailandensis* A and B) contain sliding window coverage plots that compare the coverage of each genome by different library preparation method and different DNA input amount. From the figures, it can be seen that the genome coverage is remarkably similar regardless of the library preparation method (NEBNext or TruSeq). Of particular interest is that even the libraries prepared from only 10 ng of genomic DNA produced essentially the same evenness of genome coverage as the rest of the samples (Figures 2(a)–2(d), top panel). There are some differences among the data sets, however. As Table 1 shows, the number of true gaps in coverage (0%) is slightly higher for NEBNext than for TruSeq libraries, while the number of gaps is lower for NEBNext when using 1% or 10% average coverage thresholds. The data were not normalized among all samples prior to evenness of coverage comparisons. Instead, they were normalized within each sample relative to the average coverage.

**Figure 3:**
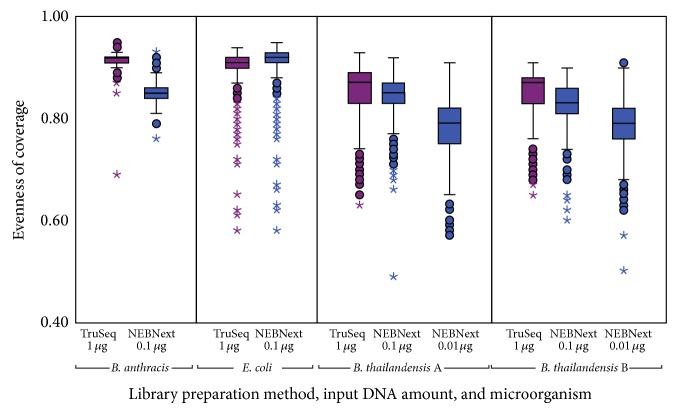
Box and whisker plots of the variation of coverage when mapping reads to the reference genomes. Variation was calculated for nonoverlapping 10 kbp windows. Evenness of coverage is calculated as 1 − (standard deviation of coverage/median coverage).

Figure 3 shows box and whisker plots of the evenness of coverage of 10 kbp windows for each genome. In the case of* E. coli*, the evenness of coverage for NEBNext Ultra libraries prepared with 100 ng of input DNA is superior to that of TruSeq libraries produced with 1 *μ*g of input DNA. For* B. anthracis *and* B. thailandensis*, there is more variation in coverage for the NEBNext preparations at both 100 ng and 10 ng. Further examination of this effect suggests that it is proportional to the amount of input DNA, supporting the theory that NEBNext Ultra kits either do not introduce bias or introduce similar bias to TruSeq kits, with a lower DNA requirement.

### 3.3. Genome Assembly

**Figure 4:**
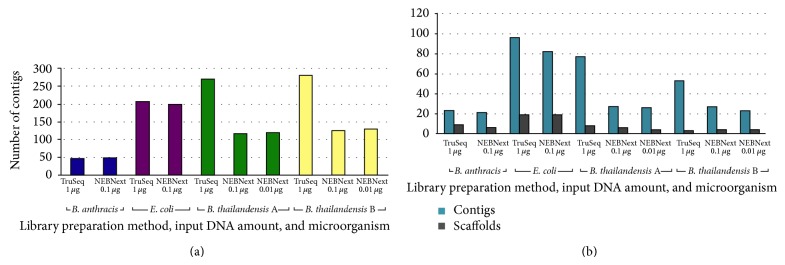
(a) IDBA assemblies of bacterial genomes using only Illumina short insert paired data. (b) Allpaths assemblies of bacterial genomes using Illumina short insert paired data and 454 long insert paired data.

Figure 4 shows the results of* de novo* genome assemblies generated using the short fragment data either alone (Figure 4(a), assembled with IDBA) or complemented with long insert mate pair data (Figure 4(b), assembled with Allpaths). IDBA assemblies show very similar results for low (*B. anthracis*) to medium (*E. coli*) %GC genomes. However, data obtained from NEBNext libraries show a dramatic reduction in the number of contigs for high %GC genomes from the two* Burkholderia* strains. Importantly, NEBNext libraries prepared from just 10 ng of genomic DNA maintain the high quality of genome assembly, producing similar numbers of contigs as with 100 ng input DNA samples. Allpaths assemblies show very similar results in terms of the number of contigs produced. The number of scaffolds does not seem to depend on the library preparation method. However, scaffolding mostly depends on the long insert mate pair data, which are the same for all assemblies. Comprehensive assembly statistics are shown in Tables S2a and S2b.

In conclusion, we have demonstrated that the quality of bacterial genome resequencing and* de novo* assembly is similar, regardless of the library preparation method and input DNA amount (from 10 to 1000 ng). The only significant difference was observed in the assemblies of the* B. thailandensis* genomes, where NEBNext library data produced dramatically more contiguous assemblies (Figure 4). In general, the assemblies from the TruSeq libraries were more prone to indels and rearrangements. The full results for the comparisons of our assemblies to the reference genomes are found in Table 2. This is likely due to the improved ability of the NEBNext reagents to more effectively amplify high %GC regions that are very common in* Burkholderia* genomes. Modern library preparation methods for next generation sequencing technologies, as represented by NEBNext Ultra, are enabling bacterial genome sequencing from very small input amounts of genomic DNA. These methods likely do not require any additional improvement, since handling and quantifying DNA amounts smaller than 10 ng can become challenging.

## 4. Availability

We have deposited the genomes of* Burkholderia thailandensis* A (strain E254, accession numbers CP004381 and CP004382) and* Burkholderia thailandensis* B (strain USAMRU Malaysia #20, accession numbers CP004383 and CP004384) into GenBank. The genome sequences will become available in the Spring of 2014.

All experimental and computational methods used during this work are publically available or can be provided by the authors.

## Supplementary Material

Supplementary data describe the NEBNext Ultra workflow (Figure S1), statistics for all NEBNext and TruSeq libraries (Table S1), and statistics for all assemblies generated with IDBA (Table 2a) and Allpaths (Table 2a) algorithms.

## Figures and Tables

**Table 1:** Comparison of gap counts by organism, replicon, and library preparation method. Input DNA amount for each sample follows the name of the library preparation method.

Gap counts for *E. coli* genome					
Library preparation method-DNA input	Reference	Replicon length, bp	Cutoff based on average coverage		
			0	1%	10%
NEBNext-100 ng	Plasmid 1	109,274	0	0	24
	Plasmid 2	74,213	45	145	318
	Plasmid 3	1,549	0	0	8
	Chromosome	5,253,138	26	206	1262
TruSeq-1000 ng	Plasmid	109,274	0	1	49
	Plasmid	74,213	44	200	491
	Plasmid	1,549	0	0	25
	Chromosome	5,253,138	23	449	2833

Gap counts for *B. thailandensis* A genome					
Library preparation method-DNA input	Reference	Replicon length, bp	Cutoff based on average coverage		
			0	1%	10%

NEBNext-10 ng	Chromosome 1	3,805,980	47	72	329
	Chromosome 2	2,870,750	86	125	498
NEBNext-100 ng	Chromosome 1	3,805,980	3	35	669
	Chromosome 2	2,870,750	78	151	1625
TruSeq-1000 ng	Chromosome 1	3,805,980	0	153	2747
	Chromosome 2	2,870,750	30	135	1937

Gap counts for *B. thailandensis* B genome					
Library preparation method-DNA input	Reference	Replicon length, bp	Cutoff based on average coverage		
			0	1%	10%

NEBNext-10 ng	Chromosome 1	3,805,980	37	158	1297
	Chromosome 2	2,870,750	19	38	717
NEBNext-100 ng	Chromosome 1	3,805,980	0	0	0
	Chromosome 2	2,870,750	6	158	2328
TruSeq-1000 ng	Chromosome 1	3,805,980	0	114	1874
	Chromosome 2	2,870,750	0	28	1177

**Table 2:** Genome assembly statistics and comparison to reference genomes for insertions/deletions and rearrangements.

Organism	Assembly method	Library preparation method	Starting DNA quantity, ng	# of contigs	Total bps	Relative to the reference genomes			
						Deletions	Insertions	Total indels	Rearrangements
*B*. *anthracis *	Allpaths	NEBNext	100	21	5,358,817	170	6,518	6,688	80
		TruSeq	1000	23	5,348,296	170	50,762	50,932	92
	IDBA	NEBNext	100	48	5,349,320	1,525	16,827	18,352	102
		TruSeq	1000	41	5,483,709	87,019	1,838	88,857	104

*E*. *coli *	Allpaths	NEBNext	100	82	5,312,046	758	48,753	49,511	222
		TruSeq	1000	96	5,269,849	760	80,227	80,987	238
	IDBA	NEBNext	100	199	5,216,101	111	152,190	152,301	414
		TruSeq	1000	207	5,242,710	224	75,926	76,150	446

*B*. *thailandensis* A	Allpaths	NEBNext	10	26	6,652,405	100	12,213	12,313	80
		NEBNext	100	27	6,650,888	143	13,042	13,185	154
		TruSeq	1000	77	6,603,636	6,294	27,237	33,531	252
	IDBA	NEBNext	10	119	6,575,406	206	38,419	38,625	276
		NEBNext	100	117	6,575,500	351	38,382	38,733	280
		TruSeq	1000	271	6,582,658	8,320	35,337	43,657	586

*B*. *thailandensis* B	Allpaths	NEBNext	10	23	6,660,010	144	12,576	12,720	82
		NEBNext	100	27	6,651,385	163	12,278	12,441	86
		TruSeq	1000	53	6,655,446	157	16,517	16,674	204
	IDBA	NEBNext	10	129	6,579,849	749	45,201	45,950	264
		NEBNext	100	125	6,579,749	236	44,279	44,515	262
		TruSeq	1000	281	6,587,931	8,694	45,997	54,691	612
