# Supplementary material for: Facile, High Quality Sequencing of Bacterial Genomes from Small Amounts of DNA
Source: Int J Genomics. 2014 Nov 13;2014:434575. doi: 10.1155/2014/434575 (PMC4247979; doi:10.1155/2014/434575)
Supplement: Supplementary file 1 — Supplementary data describe the NEBNext Ultra workflow (Figure S1), statistics for all NEBNext and TruSeq libraries (Table S1), and statistics for all assemblies generated with IDBA (Table 2a) and Allpaths (Table 2a) algorithms. [file 434575.f1.pdf]

Figure S1. NEBNext work flow.

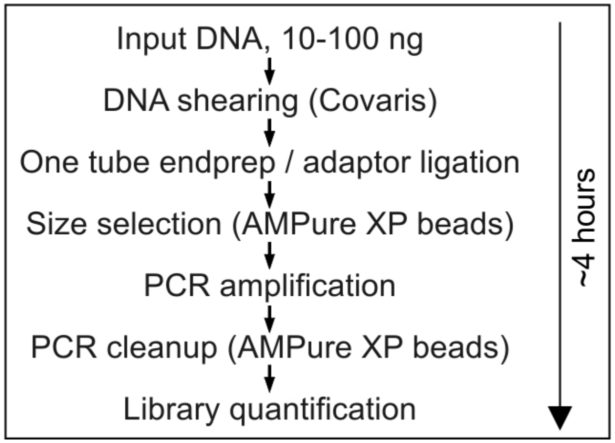

Table S1. Data statistics for all libraries.

Organism	Library prep method	% GC	Starting DNA quantity, ng	PCR cycles	Library concentration, ng/uL	Average fragment size	Library concentration, nM	Total reads	Reads with quality >Q20	Raw read length, bp	Total reads after trimming and normalization	Reads with quality >Q20 after trimming and normalization	Average read length after trimming, bp
B. anthracis	NEBNext	35.4	100	8	14.6	402	55.0	3,240,764	3,046,379	151	2,459,460	2,352,017	148
E. coli	NEBNext	50.5	100	8	11.5	403	43.0	9,079,108	8,848,462	151	2,520,000	2,457,774	151
B. thailandensis A	NEBNext	67.6	10	12	11.0	373	44.5	8,446,330	7,277,554	151	3,234,482	2,813,856	146
B. thailandensis A	NEBNext	67.6	100	10	46.5	404	174.4	9,478,982	7,961,996	151	3,168,918	2,689,132	148
B. thailandensis B	NEBNext	67.6	10	12	12.6	383	49.8	7,722,232	6,594,365	151	3,190,476	2,748,669	147
B. thailandensis B	NEBNext	67.6	100	10	47.6	370	194.9	6,241,068	5,202,194	151	3,142,858	2,665,947	147
B. anthracis	TruSeq	35.4	1000	10	59.1	248	440.7	47,836,056	19,122,933	151	2,459,460	2,362,676	149
E. coli	TruSeq	50.5	1000	10	59.4	299	237.2	18,539,690	17,101,687	151	2,571,428	2,393,342	147
B. thailandensis A	TruSeq	67.6	1000	10	55.4	429	80.3	26,415,536	13,462,977	151	3,147,652	2,104,314	149
B. thailandensis B	TruSeq	67.6	1000	10	39.9	408	40.3	41,077,890	12,813,938	101	4,690,000	2,881,288	101

Table 2a. Statistics for IDBA assemblies of Illumina short-insert data

Sample	Library Prep Kit	Starting DNA, µg	Short-insert coverage	# of contigs >1kb	# of contigs > 50kb	# of contigs > 100kb	N50 for contigs > 1kb (bp)	Largest contig (bp)	Smallest contig (bp)	Assembly Size (bp)
**B. anthracis**	Tru-Seq	1	69.0x	46	25	16	256,006	889,847	1,130	5,347,313
**B. anthracis**	NEBNext	0.1	70.0x	48	25	16	256,006	889,847	1,355	5,349,320
**E. coli**	Tru-Seq	1	61.2x	207	33	16	88,517	300,928	1,003	5,242,710
**E. coli**	NEBNext	0.1	70.4x	199	35	15	88,517	300,948	1,003	5,216,101
**B. thailandensis A**	Tru-Seq	1	70.5x	271	39	8	43,887	160,378	1,001	6,582,658
**B. thailandensis A**	NEBNext	0.1	70.3x	117	50	29	121,177	338,233	1,001	6,575,500
**B. thailandensis A**	NEBNext	0.01	70.2x	119	48	27	126,565	338,233	1,001	6,575,406
**B. thailandensis B**	Tru-Seq	1	70.6x	281	35	9	44,682	215,045	1,001	6,587,931
**B. thailandensis B**	NEBNext	0.1	70.1x	125	46	22	107,215	432,614	1,001	6,579,749
**B. thailandensis B**	NEBNext	0.01	69.1x	129	40	20	113,254	432,614	1,001	6,579,749

Table 2b. Statistics for Allpaths assemblies of Illumina short-insert data and 454 long-insert data

Sample	Library Prep Kit	Starting DNA, µg	Short-insert coverage	Long-insert coverage	Scaffolds	Number of contigs >1kb	Number of contigs > 50kb	Number of contigs > 100kb	N50 contigs > 1kb (bp)	Largest contig (bp)	Smallest contig (bp)	Assembly Size (bp)
**B. anthracis**	Tru-Seq	1	69.0x	6.8x	9	23	14	10	831,258	2,508,405	5,048	5,348,296
**B. anthracis**	NEBNext	0.1	68.7x	6.8x	6	21	13	9	996,207	2,306,221	5,048	5,358,817
**E. coli**	Tru-Seq	1	61.2x	3.4x	19	96	29	19	148,132	384,555	1,045	5,269,849
**E. coli**	NEBNext	0.1	70.4x	3.4x	19	82	30	19	180,092	625,412	1,012	5,312,046
**B. thailandensis A**	Tru-Seq	1	70.5x	7.9x	8	77	44	23	148,050	375,576	1,074	6,603,636
**B. thailandensis A**	NEBNext	0.1	70.2x	7.9x	6	27	18	15	559,003	1,096,991	1,548	6,650,888
**B. thailandensis A**	NEBNext	0.01	70.3x	7.9x	4	26	18	14	541,453	1,082,481	1,179	6,652,405
**B. thailandensis B**	Tru-Seq	1	70.6x	7.7x	3	53	35	24	206,108	575,396	2,863	6,655,446
**B. thailandensis B**	NEBNext	0.1	69.2x	7.5x	4	27	23	21	343,287	710,311	1,179	6,651,385
**B. thailandensis B**	NEBNext	0.01	70.1x	7.7x	4	23	17	16	559,910	924,997	1,897	6,660,010
